# Consistency of recommendations for pharmacotherapy of rheumatoid arthritis

**DOI:** 10.3389/fphar.2022.967787

**Published:** 2022-10-25

**Authors:** Yue Hu, Yunze Han, Yan Ma, Shumei Fan, Xue Wang, Xinyu Fu, Xiaopeng Hu, Xufei Luo, Yanfang Ma, Yangqin Xun, Nan Yang, Chengping Wen, Wei Cao, Xuping Song, Yaolong Chen

**Affiliations:** ^1^ School of Public Health, Lanzhou University, Lanzhou, China; ^2^ School of Stomatology, Lanzhou University, Lanzhou, China; ^3^ Evidence-Based Medicine Center, School of Basic Medical Sciences, Lanzhou University, Lanzhou, China; ^4^ School of Chinese Medicine, Hong Kong Baptist University, Hong Kong, Hong Kong SAR, China; ^5^ Zhejiang University of Traditional Chinese Medicine, Hangzhou, Zhejiang, China; ^6^ Wangjing Hospital, China Academy of Chinese Medical Sciences, Beijing, China; ^7^ Evidence Based Social Science Research Center, Health Technology Assessment Center, School of Public Health, Lanzhou University, Lanzhou, China; ^8^ Key Laboratory of Evidence Based Medicine and Knowledge Translation of Gansu Province, Lanzhou, China; ^9^ Institute of Health Data Science, Lanzhou University, Lanzhou, China; ^10^ WHO Collaborating Centre for Guideline Implementation and Knowledge Translation, Lanzhou, China; ^11^ Guideline International Network Asia, Lanzhou, China

**Keywords:** rheumatoid arthritis, guidelines, recommendation, consistency, strength of recommendation

## Abstract

**Background:** Rheumatoid arthritis (RA) is a chronic autoimmune inflammatory arthropathy. Recommendations for RA, specifically on pharmacotherapy, are essential in clinical practice. However, the direction and strength of recommendations are controversial across current clinical practice guidelines (CPGs) of RA.

**Objective:** To systematically analyze the consistency of recommendations regarding pharmacotherapy of RA across CPGs.

**Methods:** 11 electronic databases and websites were comprehensively searched from inception to 14 March 2022, to identify CPGs for diagnosis, therapy, and management of RA. Unambiguous and discrete specifications of the population-intervention-comparison (PIC) framework were used to classify the recommendations. Based on the PIC framework, consistency analyses across CPGs on pharmacotherapy of RA were performed. Two researchers reached a consensus on coding the direction and strength of each recommendation.

**Results:** Finally, 26 CPGs were included in this study, and 14 of them, which included pharmacotherapy, were performed consistency analysis. 1) 64 recommendations from 14 CPGs were classified into 18 PICs. 2) Seven PICs (38%) were consistent in direction and strength, 10 PICs (56%) were consistent in direction but inconsistent in strength, and one PIC (6%) was inconsistent in direction (hydroxychloroquine, HCQ). 3) Sensitivity analysis tested the robustness, and the inconsistency remained high.

**Conclusion:** The direction was highly consistent among the recommendations of pharmacotherapy for RA, but the strength was highly inconsistent. Reasons for the inconsistency need to be further investigated, and consistent recommendations could guide the pharmacotherapy of RA in clinical practice.

## 1 Introduction

Rheumatoid arthritis (RA) is a common chronic disease that manifests as a systemic autoimmune inflammatory type and involves the joints of the hands, wrists, feet, and knees (Zhang et al., 2020). RA primarily causes pain, swelling, stiffness and functional limitation of the joints but also extra-articular manifestations of other organs, including the lungs, heart, blood vessels, skin, and eyes (Smolen et al., 2016). People of all ages could be affected by RA, and the high-risk population ranges from 50 to 59 years old (Smith and Berman, 2022). RA may lead to permanent damage and disability of the joints, and it is a severe public health problem globally and carries an unbearable burden of disease. RA negatively impacts the daily quality of life in patients (Gao et al., 2022). In 2017, RA accounted for 3.4 million (95% uncertainty interval (UI), 2.6–4.4) disability adjusted life years (DALYs) at the global level, with an age-standardized rate of 43.3 (95% UI 33.0–54.5) DALYs per 1,00,000 populations (Safiri et al., 2019). RA is regarded as one of the fifty typical diseases that cause disability globally, and the incidence in women is two to three times that in men (Espinoza et al., 2021; Smith and Berman, 2022).

Clinical Practice Guidelines (CPGs) are used to provide a basis for clinical decision-making and guide clinical practice in the medical and healthcare fields. CPGs play a significant role in regulating healthcare practices, improving patient prognosis, and saving healthcare resources (Zhang et al., 2021). Recommendations formatted for specific issues are the core of the CPGs (Institute of Medicine Committee on Standards for Developing Trustworthy Clinical Practice G Graham et al., 2011). CPGs are developed based on evidence from systematic reviews, balancing the advantages and disadvantages of different interventions to form recommendations. With the growing RA research, CPGs for RA have been developed by a series of academic institutions or organizations, including the American College of Rheumatology (ACR), the European League Against Rheumatism (EULAR), and the Asia-Pacific League of Associations for Rheumatology (APLAR) (Lau et al., 2018; Ho et al., 2019; Park et al., 2020; Smolen et al., 2020). These RA CPGs are updated periodically to improve their applicability and support high-quality clinical care. The common drugs of RA therapy include disease-modifying antirheumatic drugs (DMARDs), non-steroidal anti-inflammatory drugs (NSAIDs), and glucocorticoids (GCs) (Cardiel et al., 2018; Kerschbaumer et al., 2020; Fraenkel et al., 2021). However, controversial pharmacotherapy recommendations are provided in different CPGs. For example, hydroxychloroquine (HCQ) was recommended oppositely in National Institute of Health and Clinical Excellence (NICE) and Hong Kong Society of Rheumatology (HKSR) guidelines (Ho et al., 2019; NICE, 2020). The inconsistency among pharmacotherapy recommendations impedes the practical application of these recommendations and complicates the appropriate selection of CPGs in clinical practice (Erickson et al., 2017; Ge et al., 2018; Yu et al., 2020).

As RA CPGs increased rapidly, there were significant differences and contradictions among the recommendations (Yang et al., 2013; Chen et al., 2020). Therefore, systematic reviews of CPGs were conducted to analyze the consistencies in recommendations among CPGs. Most of the consistency analyses of CPGs were performed to categorize, summarize and explain the differences among recommendations or macroscopically combine the methodology or report quality assessment results (Zhang et al., 2019; Luo et al., 2021; Zhang et al., 2021; Yan et al., 2022). However, compared to traditional analyses on the consistency of CPGs’ recommendations, the population-intervention-comparison (PIC) framework is more scientific and transparent (Alper et al., 2019; Kow et al., 2021). Currently, there is a lack of evidence on the consistency analysis of pharmacotherapy recommendations in RA CPGs (Yu et al., 2020). Therefore, we conducted a consistency analysis for RA pharmacotherapy recommendations to map the various topics covered by current RA CPGs and investigate the consistency in direction and strength among RA pharmacotherapy recommendations.

## 2 Materials and methods

### 2.1 Search strategy

We systematically searched five databases, including PubMed, DynaMed, UTD (UpToDate), CNKI (China National Knowledge Infrastructure), and CBM (China Biology Medicine). Additionally, WHO (World Health Organization), GIN (Guidelines International Network), NICE (National Institute for Health and Care Excellence), SIGN (Scottish Intercollegiate Guidelines Network), ECRI (Emergency Care Research Institute), and Medlive were searched. Our comprehensive search was limited from inception to 14 March 2022. The search strategy consisted of keywords: (“rheumatoid arthritis”) AND (“guidance” OR “guideline” OR “recommendation”). The detailed search strategy was shown in Supplementary Table S1.

### 2.2 Inclusion and exclusion criteria

Inclusion criteria: 1) guidelines for the diagnosis, treatment, and management of RA; 2) the latest and available version of the guidelines. Exclusion criteria: 1) guidelines published in languages other than Chinese or English; 2) an interpretation version of the guidelines; 3) the protocol of guidelines.

### 2.3 Literature selection and data extraction

Two researchers independently screened the titles and abstracts to exclude irrelevant literature. The literature, which could not be determined by screening the titles and abstracts, needed to be identified by reviewing the full text. Data extraction was conducted using pre-designed tables in Microsoft Excel 2016. The extracted items included the title, publication year, publication language, development institution/organization, type of guideline, the adaption of other guidelines or not, the method for grading the quality of evidence and strength of recommendations, and counts of recommendations. Data extraction was carried out by two researchers independently. If there were disagreements between literature selection and data extraction, a third researcher was consulted to resolve the disagreements by reaching a consensus.

### 2.4 Consistency analysis

#### 2.4.1 Inclusion and exclusion criteria of the consistency analysis

We selected guidelines for the consistency analysis from the included studies. Inclusion criteria were 1) guidelines that contained recommendations for pharmacotherapy. Exclusion criteria were 1) guidelines adapted from other guidelines; 2) traditional Chinese medicine (TCM) guidelines; 3) guidelines that the counts of pharmacotherapy recommendations were not stated in the abstracts or full text, which made it impossible to extract.

#### 2.4.2 Recommendation specification

We identified the recommendations regarding pharmacotherapy of RA in the selected CPGs in consistency analysis. Population-intervention-comparison (PIC) concepts precluded differentia among recommendations compared to simple and direct consistency analysis. “P” means population or patient, which should describe the recommendation as appropriate for who or under what circumstances. “I” means intervention, which should describe specifically, such as monotherapy, therapy combination, therapy switch, and dosage reduction. “C” means comparison, which should describe the specific pharmacotherapy compared to the intervention. PIC framework was used to compare the recommendations with the same PIC descriptions. All recommendations about pharmacotherapy of RA were looked through to confirm counts of PIC. Only when two or more recommendations described the same PIC, can the recommendations be compared and formed a PIC. Only one recommendation had an independent PIC, which means no recommendation could compare with it, would be excluded. We included as many comparable recommendations as possible by separating PIC items without changing the original meanings of recommendations. Two researchers reached an agreement in the process. Any disagreements were discussed and solved with a third party. Recommendation specification was a key step that could disambiguate the difference among recommendations.

#### 2.4.3 Coding direction and strength of recommendations

We assessed the recommendations of each CPG in three steps. Firstly, we assessed whether the CPG contained the recommendation within the range of PICs. If not, we classified the CPG as “out of scope,” and no further coding application was used. Secondly, we assessed the direction of recommendation by comparing the comparison and the intervention. We classified the CPG as “recommended” if it recommended the intervention over the comparison. Conversely, we classified the CPG as “not recommended” if the comparison was recommended over the intervention. Thirdly, the strength of recommendation was assessed. If the recommendation was rated as strong, “A” grade, the highest degree of the strength, or used definitive language to describe the highest degree of obligation or expectations for following the recommendation, we coded the recommendation as “strong strength.” If the recommendation was rated as less than the highest degree of the strength or used indeterminate language to describe a lower degree of obligation or expectations for following the recommendation, we coded the recommendation as “weak strength.” The recommendation without the strength or the CPG without the method for grading the quality of evidence and strength of recommendations, we coded the recommendation as “unspecified strength.”

### 2.5 Sensitivity analysis

Firstly, recommendations with unspecified strength or guidelines without using a method for grading the quality of evidence and strength of recommendations were excluded from viewing the changes in the direction and strength of recommendations. Secondly, leave-one-out sensitivity analyses were used to assess the robustness of consistency after excluding one guideline at a time, looking at changes in the direction and strength of each PIC in turn. Sensitivity analysis was carried out through these two steps to check whether the scope of included guidelines in the consistency analysis and the strength of the recommendation has a clear impact on the direction and strength of the recommendation and the fluctuation range of the consistency rate.

## 3 Results

### 3.1 Characteristics of included clinical practice guidelines

**FIGURE 1:**
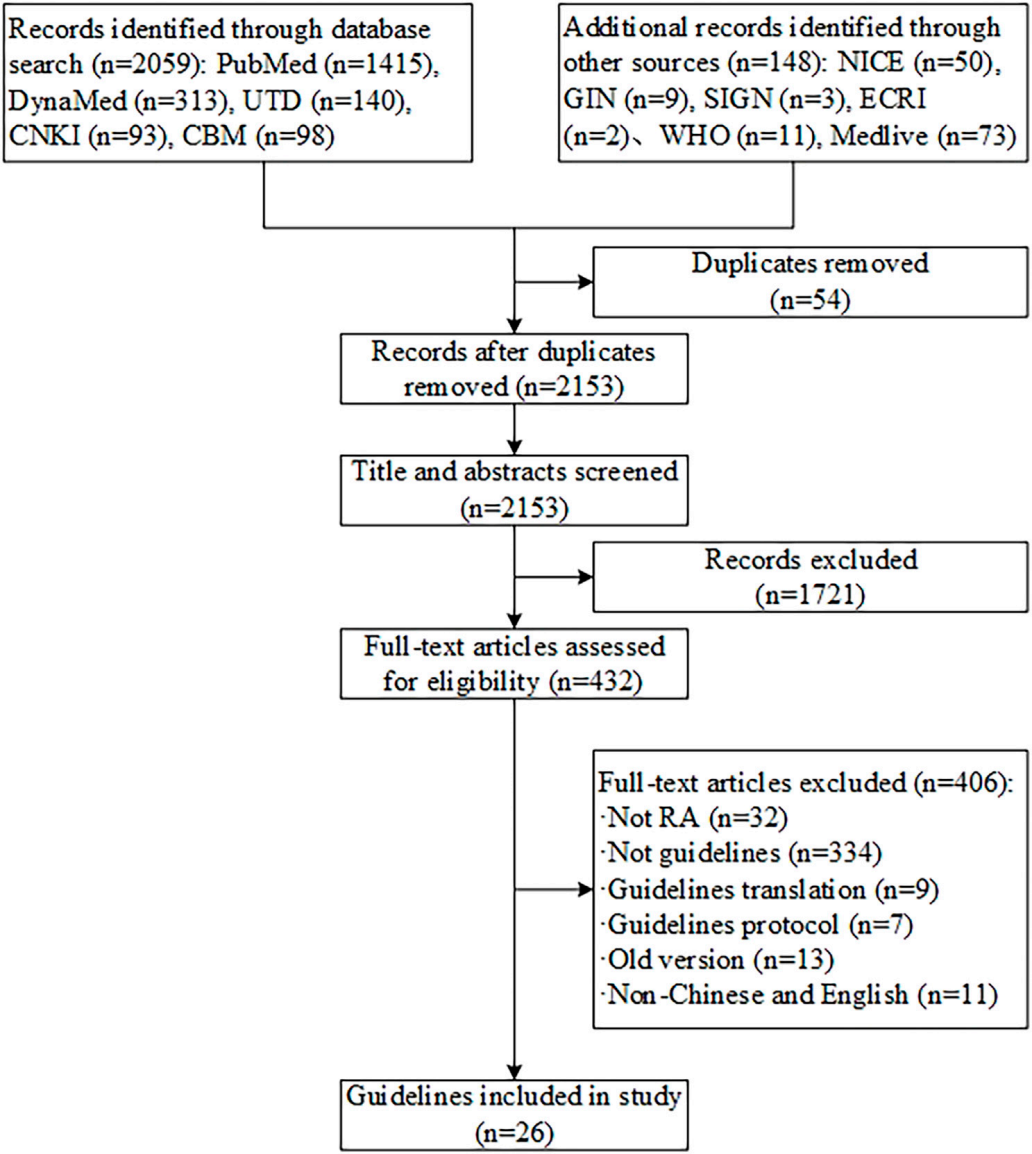
Flow diagram of the literature screening process.

We initially identified 2,207 records, 54 duplicates removed by Endnote software, 1,721 records were excluded after the full-text screening, and 406 records were excluded by reviewing the full text. Ultimately, 26 guidelines met the criteria (see Figure 1). (Lau et al., 2018; Smolen et al., 2020; Ho et al., 2019; Fraenkel et al., 2021; Cardiel et al., 2018; NICE, 2020; Peter et al., 2021; Fang et al., 2020; Alhajeri et al., 2019; Daien et al., 2019; Parisi et al., 2019; Leon et al., 2018; Ataman et al., 2018; Chinese Rheumatology, 2018; Henrique da Mota et al., 2012; Hurkmans et al., 2011; Forestier et al., 2009; Gossec et al., 2006; Jiang et al., 2018; Huang et al., 2011; Jiang, 2019-10; Guidelines for diagnosis and treatment of rheumatoid arthritis, 2010; Luo et al., 2020; Management of Rheumatoid Arthritis, 2019; Management of early rheumatoid arthritis, 2011; Clinical guideline for the diagnosis and management of early rheumatoid arthritis, 2009) Twenty-six guidelines were included in publication years ranging from 2006 to 2021. Eight CPGs were developed in China, three in France, two in the Netherlands, and one in each of the United States, Mexico, the United Kingdom, Europe, Asia Pacific, Italy, Malaysia, Brazil, Scotland, Australia, Spain, Kuwait, and Turkey. The types of included guidelines consisted of diagnosis, treatment, and management. The specific types of therapy were pharmacotherapy, physiotherapy, TCM therapy, psychotherapy, and rehabilitation therapy. The methods for grading the quality of evidence and the strength of recommendations were different. Seven CPGs used the Grading of Recommendations, Assessment, Development, and Evaluations (GRADE), two used the adapted GRADE, three used the Oxford Centre for Evidence-Based Medicine (OCEBM) criteria, one used the Scottish Intercollegiate Guidelines Network (SIGN), one used the Evidence-Based Recommendation Development (EBRO), one used the National Health and Medical Research Council (NHMRC), and four adopted other unnamed standards or tools. The method for grading the quality of evidence and strength of recommendations was not reported in one CPG, and six CPGs not used (see Supplementary Table S2).

### 3.2 Consistency analysis

**FIGURE 2:**
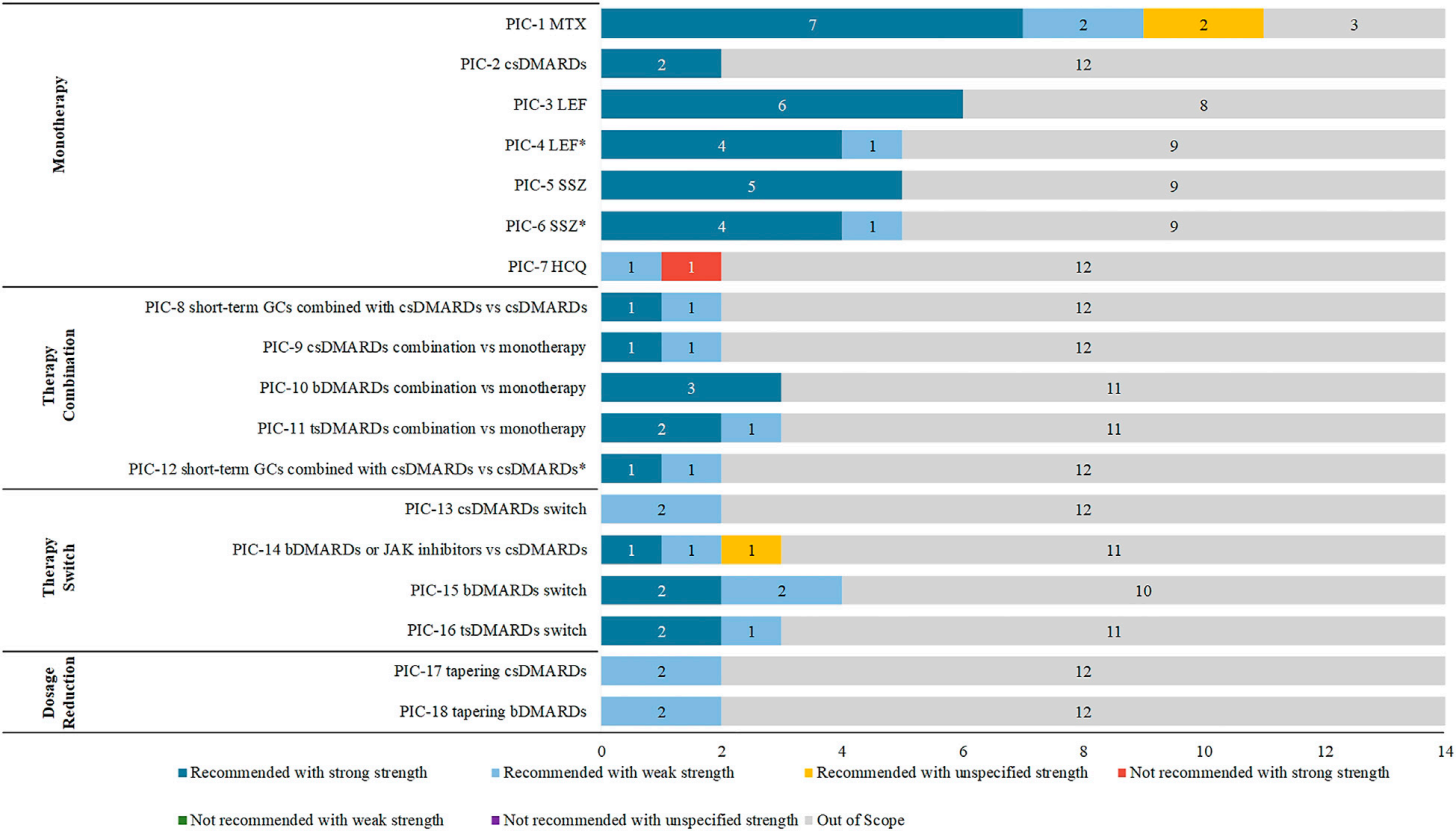
Consistency analysis of 18 PICs across 14 CPGs.

We selected 14 guidelines for consistency analysis from the included guidelines, with the number of recommendations ranging from 10 to 47 (see Supplementary Table S2). The publish year of 11 (78.57%, 11/14) guidelines ranged from 2018 to 2021. Other three guidelines (RACGP, SIGN, and BSR) were published in 2009, 2011, and 2012 respectively. Sixty-four recommendations of the 14 included guidelines were extracted and analyzed. After PIC coding, 18 PICs were obtained (see Table 1), and the details of the direction and strength of the recommendations were shown in Supplementary Table S3. There were 17 (17/18, 94%) PICs with consistent direction, and the only PIC (1/18, 6%) with an inconsistent direction was HCQ monotherapy. There were 7 (7/18, 38%) PICs with consistent direction and strength, including csDMARDs monotherapy, leflunomide (LEF) monotherapy or salazosulfapyridine (SSZ) monotherapy when methotrexate (MTX) was contraindicated, csDMARDs in combination with bDMARDs, and dosage reduction of csDMARDs and bDMARDs (see Figure 2). Among the 18 PICs, the direction of the recommendations was highly consistent, but poor consistency was found in the strength of the recommendations.

**TABLE 1:** Population-intervention-comparator specifications.

Code	Name	Population	Intervention	Comparator
PIC-1	MTX	As soon as the diagnosis of RA	MTX	No MTX
PIC-2	csDMARDs	Adult patients who newly diagnosed active RA	csDMARDs monotherapy	No csDMARDs
PIC-3	LEF	Patients who have contraindication to MTX	LEF	MTX
PIC-4	LEF	Patients who have intolerance to MTX	LEF	MTX
PIC-5	SSZ	Patients who have contraindication to MTX	SSZ	MTX
PIC-6	SSZ	Patients who have intolerance to MTX	SSZ	MTX
PIC-7	HCQ	Adult patients who newly diagnosed active RA	HCQ	No HCQ
PIC-8	Short-term GCs combined with csDMARDs vs. csDMARDs	Patients who start new csDMARDs therapy	Short-term GCs combined with csDMARDs	csDMARDs
PIC-9	csDMARDs combination vs. csDMARDs monotherapy	Patients who treatment target is not achieved with monotherapy csDMARDs	csDMARDs combination	csDMARDs monotherapy
PIC-10	bDMARDs combination vs. bDMARDs monotherapy	Patients who treatment target is not achieved with the first csDMARDs strategy and poor prognostic factors are present	bDMARDs combination	csDMARDs monotherapy
PIC-11	tsDMARDs combination vs. tsDMARDs monotherapy	Patients who treatment target is not achieved with the first csDMARDs strategy and poor prognostic factors are present	tsDMARDs combination	csDMARDs monotherapy
PIC-12	Short-term GCs combined with csDMARDs vs. csDMARDs	Patients who moderate-to-high disease activity	Short-term GCs combined with csDMARDs	csDMARDs
PIC-13	csDMARDs switch	Patients who treatment target is not achieved with csDMARDs and in the absence of poor prognostic factors	Other csDMARDs	Same csDMARDs
PIC-14	bDMARDs or JAK inhibitors vs. csDMARDs	Patients who treatment target is not achieved with moderate-to-high disease activity	bDMARDs or JAK inhibitors	csDMARDs
PIC-15	bDMARDs switch	Patients who treatment target is not achieved with bDMARDs	Other bDMARDs	Same bDMARDs
PIC-16	tsDMARDs switch	Patients who treatment target is not achieved with tsDMARDs	Other tsDMARDs	Same tsDMARDs
PIC-17	Tapering csDMARDs	Patients in sustained remission	Tapering csDMARDs	Not tapering csDMARDs
PIC-18	Tapering bDMARDs	Patients in sustained remission	Tapering bDMARDs	Not tapering bDMARDs

MTX: methotrexate; LEF: leflunomide; SSZ: sulfasalazine; HCQ: hydroxychloroquine; GCs: glucocorticoids; csDMARDs: conventional synthetic disease modifying anti-rheumatic drugs; bDMARDs: biologic disease modifying anti-rheumatic drugs; tsDMARDs: targeted synthetic disease modifying anti-rheumatic drugs.

#### 3.2.1 Monotherapy

In the monotherapy dimension (PIC1-7), csDMARDs such as MTX, LEF, SSZ, and HCQ were included. Among these PICs, the most recommendations were about MTX and had been included from 11 (11/14, 78.57%) CPGs with the same direction. Further, seven of them had strong strength of recommendation, two had weak strength of recommendation, and two had unspecified strength of recommendation. The two recommendations for csDMARDs monotherapy were consistent in direction and strength. The direction of LEF and SSZ were consistent, but their strengths were diverse when used under different conditions. When MTX was contraindicated, either LEF or SSZ was strongly recommended; when MTX was not tolerated, EULAR, HKSR, SIR, and SFR guidelines gave strong recommendations for LEF or SSZ, and APLAR guidelines gave weak strength of recommendation. The recommendations for HCQ were in different directions. The NICE guideline indicated that HCQ should be considered a first-line treatment (weak strength of recommendation). In contrast, HKSR guideline explained that HCQ should not be used as a first-line treatment for RA (strong strength of recommendation).

#### 3.2.2 Therapy combination

In the therapy combination dimension (PIC8-12), PICs included csDMARDs combined with short-term GCs, csDMARDs combination, bDMARDs combination, or tsDMARDs combination, and their directions were consistent. PIC8 and 12 were both csDMARDs combined with short-term GCs, but the population and strength of the recommendations were inconsistent. The population of PIC8 was patients who started new csDMARDs therapy, which was weak strength of recommendation in NICE guidelines and strong strength of recommendation in EULAR guidelines. In contrast, the population of PIC12 was patients who had moderate-to-high disease activity, which was strong strength of recommendation in SIR guideline and weak strength of recommendation in CRA guideline. The combination of csDMARDs (PIC9) had different strengths. It was strongly recommended in HKSR guideline but weakly in CRA guideline. The direction and strength of the recommendations for csDMARDs combined with bDMARDs (PIC10) were consistent and strong recommendations were given by EULAR, HKSR, and SIR guidelines. In the PIC11, tsDMARDs combination, three CPGs (EULAR, HKSR, and SIR guidelines) gave strong recommendations but only weak CRA guideline.

#### 3.2.3 Therapy switch

In the therapy switch dimension (PIC13-16), the therapy switch was considered when the current treatment strategy failed to achieve the goal, which included the switch about csDMARDs, bDMARDs, and tsDMARDs. The strength of recommendations was inconsistent in all PICs (PIC14-16) except for the PIC13 of another csDMARDs should be considered when the csDMARDs treatment goal failed, which EULAR and SIR guidelines recommended with a weak strength of recommendation. The switch to bDMARDs or JAK inhibitors (PIC14) had included three recommendations from CMR, APLAR, and MOH Malaysia guidelines with different strengths, strong, weak and unspecified. The PIC15, switch to bDMARDs, included four recommendations. Two of them had strong strength of recommendation (EULAR and SFR guidelines), and two had weak strength of recommendation (ACR and HKSR guidelines). The switch to tsDMARDs (PIC16) included three recommendations strongly recommended in EULAR and SFR guidelines but weakly in ACR guideline.

#### 3.2.4 Dosage reduction

In the dosage reduction dimension (PIC17-18), csDMARDs or bDMARDs were considered to reduce the dose when patients were in sustained remission. Two recommendations were included in PIC17 and PIC18, which had the same direction and strength. In dosage reduction of csDMARDs, EULAR, and SIR guidelines were recommended with a weak strength of recommendation. In PIC18, tapering bDMARDs was weakly recommended in HKSR and SIR guidelines.

### 3.3 Sensitivity analysis

The inconsistency rate remained high after excluding recommendations with unspecified strength (see Table 2; Supplementary Table S4). The leave-one-out sensitivity analysis revealed varying degrees of variation in the consistency rate (see Supplementary Tables S5–S18). The number of coded PICs varied from 13 to 18. The variation in the direction of recommendation consistency rate ranged from -1% to 6%; the variation in the rate for both direction and strength consistency ranged from -15.8% to 14.1% (see Table 2).

**TABLE 2:** Consistency of direction and strength across clinical practice guidelines with sensitivity analysis for excluding unspecified strength.

Analysis	Number of PICs	Number (%) with consistency in direction	Number (%) with consistency in direction and strength
Overall analysis	18	17 (94.4%)	7 (38.9%)
Excluding “unspecified strength”	18	17 (94.4%)	7 (38.9%)
Excluding “ACR”*, excluding “unspecified strength”	18	17 (94.4%)	8 (44.4%)
Excluding “CMR"*, excluding “unspecified strength”	17	16 (94.1%)	7 (41.2%)
Excluding “GPCRCID”*, excluding “unspecified strength”	18	17 (94.4%)	7 (38.9%)
Excluding “NICE”*, excluding “unspecified strength"	15	15 (100%)	6 (40.0%)
Excluding “EULAR”*, excluding “unspecified strength”	15	14 (93.3%)	5 (33.3%)
Excluding “APLAR”*, excluding “unspecified strength”	17	16 (94.1%)	9 (53.0%)
Excluding “HKSR”*, excluding “unspecified strength”	15	14 (100%)	6 (40.0%)
Excluding “SIR”*, excluding “unspecified strength”	13	12 (92.3%)	3 (23.1%)
Excluding “MoH Malaysia”*, excluding “unspecified strength”	18	17 (94.4%)	7 (38.9%)
Excluding “SFR”*, excluding “unspecified strength”	18	17 (94.4%)	7 (38.9%)
Excluding “CRA”*, excluding “unspecified strength”	16	15 (93.8%)	8 (50.0%)
Excluding “BSR”*, excluding “unspecified strength”	18	17 (94.4%)	7 (38.9%)
Excluding “SIGN”*, excluding “unspecified strength”	18	17 (94.4%)	7 (38.9%)
Excluding “RACGP”*, excluding “unspecified strength”	18	17 (94.4%)	7 (38.9%)

*Unspecified strength: recommended with unspecified strength of recommendation; ACR, American college of rheumatology; CMR, Mexican college of rheumatology; GPCRCID, Guangdong provincial clinical research center for immunological diseases; NICE, national institute of health and clinical excellence; EULAR, European league against rheumatism; APLAR, Asia-Pacific League of Associations for Rheumatology; HKSR, Hong Kong society of rheumatology; SIR, Italian society for rheumatology; MoH Malaysia, Ministry of Health Malaysia; SFR, French society for rheumatology.

Here we took the CPGs which had the most significant rate changes in the leave-one-out sensitivity analysis as examples to describe. After excluding recommendations with unspecified strength, the direction consistency rate increased to 100% after NICE or HKSR guideline was excluded (see Supplementary Tables S8, S11) and decreased to 92.3% when SIR guideline was excluded (see Supplementary Table S12). After excluding APLAR guideline, the number of recommendations with consistent direction and strength increased to nine, and the consistency rate increased to 53.0% (see Supplementary Table S10). In contrast, after excluding SIR guideline, the number of recommendations with consistent direction and strength remained only three, and the consistency rate decreased to 23.1% (see Supplementary Table S12).

## 4 Discussion

This study systematically searched the diagnosis, treatment, and management CPGs of RA to map the current topics and pharmacotherapy of RA. Further, the consistency analysis was conducted for the recommendations related to RA pharmacotherapy. The results showed that the pharmacotherapy of RA was consistent in the direction, but the strength was highly inconsistent.

### 4.1 The significance of consistency analysis of rheumatoid arthritis recommendations in clinical practice guidelines

The recommendations among different CPGs create difficulties in clinical practice and decision-making, specifically when faced with inconsistent directions or various conditions related to recommendations (Mackey and Liang, 2011). In our study, HCQ was recommended in different directions. NICE guideline indicated that HCQ should be considered as a first-line treatment (NICE, 2020), but HKSR stated not to use HCQ because of its slow onset and the availability of more effective csDMARDs (Ho et al., 2019). Recommendations on the HCQ of these two guidelines were formed merely on primary studies, not systematic reviews. Based on a systematic review, HCQ in monotherapy had similar (or even lower) efficacy compared to other csDMARDs. However, the combination of HCQ and other DMARDs would increase the clinical efficacy (Rempenault et al., 2020). Therefore, guideline developers should clearly describe the evidence on which the recommendations are based, the factors considered, and the process and methods used to form the recommendations. Therefore, clinicians might face a dilemma when treating RA with pharmacotherapy. Because following the recommendations in one guideline may violate the recommendations in other guidelines. Clinicians often give special attention to the strength of evidence, but we found that the strength of recommendation in the same PIC was inconsistent. Thus, it is essential to specify the circumstances in which the pharmacotherapy of RA should be used. These issues have complicated the implementation and application of guidelines and resulted in the disconnection between evidence and clinical practice. The inconsistency in CPGs indicated that the clinical questions were still unspecific and unclear, which needed to be supported by more studies. Therefore, we advocated paying more attention to controversial recommendations and updating the guidelines in time to improve the consistency.

### 4.2 Potential reasons for poor consistency of rheumatoid arthritis recommendations

There are many underlying reasons for the poor consistency among recommendations. First, the time span of the guideline development may cause heterogeneity because the emergence of new evidence may overturn old concepts or perspectives (Kow et al., 2021; Zhang et al., 2021). Therefore, the differences among the guidelines could be observed according to the publication year. Second, the lack of high-quality evidence may lead to formulating recommendations based on expert opinions rather than evidence. Further, the selection and interpretation of evidence may be inconsistent among guidelines. Third, different countries and regions consider local conditions, factors, values, preferences, and types and degrees of stakeholder participation differently. Fourth, different countries and regions may have quite different populations and population composition, and drug policies, which might impact the development of clinical recommendations. RA imposes a substantial economic burden and the expenditures for RA pharmacotherapy increase with severity (Santos-Moreno et al., 2021). Drug policy, clinical recommendations, and consensus conferences of different countries or regions should be considered judiciously during specific applications of innovative and expensive pharmacology. Fifth, different methods for grading the quality of evidence and strength of recommendations may form inconsistent recommendations based on the same evidence (Jolliffe et al., 2018). These reasons might lead to high-inconsistent recommendations. Further research should determine the reasons for the inconsistency.

### 4.3 Study strengths and limitations

The strengths of this study were as follows. First, to our knowledge, this is the first study that used the PIC framework to analyze the consistency of RA pharmacotherapy recommendations among CPGs. Second, we used a robust scientific PIC framework to encode the recommendations and analyzed the consistency among the recommendations in detail. Third, we focused on commonly-used and essential pharmacotherapy in the clinic. We also visualized the overall consistency among these recommendations, providing concise and detailed information for clinicians and researchers who may benefit from these findings while noting the existing gaps.

This study had some limitations as well. There may be some bias as the methods for grading the quality of evidence and strength of recommendations were different. The strength of recommendations was divided into strong and weak, and the highest degree of strength was coded as strong. The strength less than the highest degree was coded as weak according to the strength given in the guidelines. Local circumstances and drug policies vary among countries and should also be noticed, which may make recommendations different and create bias.

### 4.4 Suggestions for the future rheumatoid arthritis research and clinical practice guidelines

Developers need to refer to internationally recognized methods and standards for guideline development. First, developers should write a protocol and register before the guideline development work is carried out (Ge et al., 2018). Second, forming recommendations should adopt the currently recognized and more applicable methods for grading the quality of evidence and strength of recommendations, such as GRADE (Group, 2012; Dijkers, 2013). Further, the recommendations should be based on the highest quality of evidence, and their usage conditions should be clarified (Brignardello-Petersen et al., 2021). Accordingly, CPGs could be better implemented to provide evidence-based support for clinical practice and decision-making. However, the communications between CPG developers may be confused or hindered if the grading systems of evidence are different (Brozek et al., 2009; Sun et al., 2019). Third, high-quality evidence for RA should be produced *via* original research and systematic reviews in the future to develop RA guidelines and support the recommendations. Many factors need to be considered and balanced during the development of guidelines, so it is hard to maintain a high degree of consistency in the strength of the recommendations. Forth, systematic reviews of guidelines are suggested to conduct, indicating research gaps. Future studies on the consistency of recommendations among CPGs should pay attention to the following points. First, with the CPGs updated quickly, the publish time period of CPGs need to be considered. Second, whether CPGs are national or just developed by the single institution (or organization) and should be checked. It is inappropriate to analyze these CPGs together. Third, the availability of new drugs in the nations should be scrutinized. Because the local circumstances and drug policies are various. With the continuous revising and updating of the CPGs, we believe that high-quality guidelines could be developed in the future to form clear, definite, and consistent recommendations for the pharmacotherapy of RA.

## 5 Conclusion

Our study found that current recommendations for RA pharmacotherapy were almost consistent in direction but were highly inconsistent in strength. CPGs should be standardized and scientifically developed further. High-quality original research and systematic reviews on RA pharmacotherapy with inconsistent direction and strength are needed to fill the research gaps.

## Data Availability

The original contributions presented in the study are included in the article/Supplementary Material, further inquiries can be directed to the corresponding authors.
