# Supplementary material for: Consistency of recommendations for pharmacotherapy of rheumatoid arthritis
Source: Front Pharmacol. 2022 Oct 25;13:967787. doi: 10.3389/fphar.2022.967787 (PMC9642806; doi:10.3389/fphar.2022.967787)
Supplement: Supplementary file 1 [file DataSheet1.pdf]

SUPPLEMENTARY APPENDIX

Consistency of Recommendations for Pharmacotherapy of Rheumatoid Arthritis

Summary of Supplementary data

Table S1. Search Strategy for PubMed.

Table S2. Characteristics of the Included Clinical Practice Guidelines.

Table S3. Direction and Strength Ratings by Recommendations Across Clinical Practice Guidelines Included in Consistency Analysis.

Table S4. Consistency of Direction and Strength Ratings by Recommendation Across Clinical Practice Guidelines Excluding Unspecified Strength*.

Table S5. Consistency of Direction and Strength Ratings by Recommendation Across Clinical Practice Guidelines Excluding ACR*.

Table S6. Consistency of Direction and Strength Ratings by Recommendation Across Clinical Practice Guidelines Excluding CMR*.

Table S7. Consistency of Direction and Strength Ratings by Recommendation Across Clinical Practice Guidelines Excluding GPCRCID*.

Table S8. Consistency of Direction and Strength Ratings by Recommendation Across Clinical Practice Guidelines Excluding NICE*.

Table S9. Consistency of Direction and Strength Ratings by Recommendation Across Clinical Practice Guidelines Excluding EULAR*.

Table S10. Consistency of Direction and Strength Ratings by Recommendation Across Clinical Practice Guidelines Excluding APLAR*.

Table S11. Consistency of Direction and Strength Ratings by Recommendation Across Clinical Practice Guidelines Excluding HKSR*.

Table S12. Consistency of Direction and Strength Ratings by Recommendation Across Clinical Practice Guidelines Excluding SIR*.

Table S13. Consistency of Direction and Strength Ratings by Recommendation

Across Clinical Practice Guidelines Excluding MoH Malaysia*.

Table S14. Consistency of Direction and Strength Ratings by Recommendation Across Clinical Practice Guidelines Excluding SFR*.

Table S15. Consistency of Direction and Strength Ratings by Recommendation Across Clinical Practice Guidelines Excluding CRA*.

Table S16. Consistency of Direction and Strength Ratings by Recommendation Across Clinical Practice Guidelines Excluding BSR*.

Table S17. Consistency of Direction and Strength Ratings by Recommendation Across Clinical Practice Guidelines Excluding SIGN*.

Table S18. Consistency of Direction and Strength Ratings by Recommendation Across Clinical Practice Guidelines Excluding RACGP*.

Table S1. Search Strategy for PubMed.

No.	Search Strategy
#1	"Arthritis, Rheumatoid".sh
#2	rheumatoid arthritis.ti, ab
#3	or/1,2
#4	"Guidelines as Topic".sh or Guideline.pt
#5	guidance*/or consensus*/or statement*/or recommendation*.ti
#6	or/4,5
#7	and/3,6

Table S2. Characteristics of the Included Clinical Practice Guidelines.

Code	Development institute/ organization	Publish year	Language	Country/ Region	Type of guideline	Specific type of therapy	Adaption of other guidelines	Method for evaluating the quality of evidence and strength of recommendations	Counts of recommendations
1	KNGF	2021	English	Netherlands	Therapy	Physical Therapy	N	Adapted GRADE	9
2*	ACR	2021	English	America	Therapy	Pharmacotherapy	N	GRADE	44
3*	CMR	2021	English	Mexico	Therapy	Pharmacotherapy	N	GRADE	24
4	ATHHUM	2020	Chinese	China	Therapy	TCM Rehabilitation Therapy	N	NR	Unclear
5*	GPCRCID	2020	Chinese	China	Diagnosis; Therapy; Management	Therapy	N	GRADE	16
6	WFCMS; CATCM	2020	Chinese	China	Therapy	TCM Therapy	N	GRADE	Unclear
7*	NICE	2020	English	England	Diagnosis; Therapy; Management	Therapy	N	Words indicate strength [a]	47
8*	EULAR	2020	English	Europe	Therapy	Pharmacotherapy	N	OCEBM	12
9*	APLAR	2019	English	Asia-Pacific region	Therapy	Pharmacotherapy	N	GRADE	16
10*	HKSR	2019	English	China	Therapy; Management	Therapy	N	Adapted GRADE	36
11*	SIR	2019	English	Italy	Therapy; Management	Therapy	N	OCEBM	14
12*	MoH Malaysia	2019	English	Malaysia	Diagnosis; Therapy; Management	Therapy	N	NU	10

Code	Development institute/ organization	Publish year	Language	Country/ Region	Type of guideline	Specific type of therapy	Adaption of other guidelines	Method for evaluating the quality of evidence and strength of recommendations	Counts of recommendations
13*	SFR	2019	English	France	Diagnosis; Therapy; Management	Therapy	N	A 0–10 scale [b]	15
14	KAR	2019	English	Kuwait	Therapy	Therapy	Y	NU	66
15*	CRA	2018	Chinese	China	Diagnosis; Therapy	Therapy	N	GRADE	10
16	RUISCCH	2018	English	Spain	Management	—	N	OCEBM	13
17	BCRCACM	2018	Chinese	China	Diagnosis; Therapy	TCM Therapy	N	GRADE	Unclear
18	TLAR	2018	English	Turkey	Therapy	Pharmacotherapy	Y	NU	12
19*	BSR	2012	English	Brazil	Therapy	Therapy	N	NU	20
20*	SIGN	2011	English	Scotland	Therapy; Management	Therapy	N	SIGN	17
21	CATCM	2011	Chinese	China	Diagnosis; Therapy	TCM Therapy	N	NU	Unclear
22	LUMC	2011	English	Netherlands	Therapy	Physical Therapy	N	EBRO	7
23	CRA	2010	Chinese	China	Diagnosis; Therapy	Therapy	N	NU	Unclear
24*	RACGP; NHMRC	2009	English	Australia	Diagnosis; Therapy; Management	Therapy	N	NHMRC	30
25	A multidisciplinary working group (18 experts)	2009	English	France	Therapy	Non-pharmacological Therapy	N	A three level standard [c]	33
26	A scientific committee composed of hospital-based rheumatologists	2006	English	France	Therapy	Non-pharmacological Therapy	N	A classification scheme for category of evidence and strength of recommendation [d]	5

KNGF: Royal Dutch Society for Physical Therapy; ACR: American College of Rheumatology; CMR: Mexican College of Rheumatology; ATHHUM: Affiliated Taihe Hospital of Hubei University of Medicine; GPCRCID: Guangdong Provincial Clinical Research Center for Immunological Diseases; WFCMS: World Federation of Chinese Medicine Societies; CATCM: China Association of Traditional Chinese Medicine; LUMC: Leiden University Medical Center; NICE: National Institute of Health and Clinical Excellence; EULAR: European League Against Rheumatism; APLAR: Asia-Pacific League of Associations for Rheumatology; HKSR: Hong Kong Society of Rheumatology; SIR: Italian Society for Rheumatology; MoH Malaysia: Ministry of Health Malaysia; SFR: French Society for Rheumatology; KAR: Kuwait Association of Rheumatology; CRA: Chinese Rheumatology Association; RUISCCH: Rheumatology Unit, IDISSC, San Carlos Clinical Hospital; BCRCACM: Branch Committee of Rheumatology of China Association of Chinese Medicine; TLAR: Turkish League Against Rheumatism; BSR: Brazilian Society of Rheumatology; SIGN: Scottish Intercollegiate Guidelines Network; RACGP: Royal Australian College of General Practitioners; NHMRC: National Health and Medical Research Council; TCM: Traditional Chinese Medicine; GRADE: Grading of Recommendations Assessment, Development and Evaluation; OCEBM: Oxford Centre for Evidence-Based Medicine; EBRO: Evidence Based Recommendation Development; Y: yes; N: no; NR: not reported; NU: not used.

[*] These 14 CPGs were included in the consistency analysis of recommendations between different CPGs.

[a] Words indicate strength: 'offer' means clear and strong evidence of benefit; 'consider' means the benefit is less certain.

[b] A0–10 Scale: 0 indicated complete disagreement and 10 complete agreements, the level of the underlying evidence and the grade of each recommendation were determined.

[c] A three level standard: A-Trials of a high level of evidence (level of evidence 1), e.g. high power randomized controlled trials (RCTs) free of major bias and/or meta-analyses of RCTs or decision analyses based on level 1 trials; B-Studies of an intermediate level of evidence (level of evidence 2), e.g. RCTs with some bias, meta-analyses based on questionable methodology, well-conducted non-randomized controlled trials or cohort studies; C-Studies of a lower level of evidence, e.g. case control studies (level of evidence 3) or case series (level of evidence 4).

[d] A classification scheme for category of evidence and strength of recommendation: The categories of evidence are divided into: Ia-evidence for meta-analysis of randomized controlled trials; Ib-evidence from at least one randomized controlled trial; IIa-evidence from at least one controlled study without randomization; IIb — evidence from at least one other type of quasi-experimental study; III-evidence from non-experimental descriptive studies, such as comparative studies, correlation studies, and case-control studies; IV-evidence from expert committee reports or opinions or clinical experience of respected authorities, or both. The strength of the proposal is divided into: A—directly based on category I evidence; B—directly based on category II evidence or extrapolated recommendation from category I evidence; C—directly based on category III evidence or extrapolated recommendation from category I or II evidence; D—directly based on category IV evidence or extrapolated recommendation from category I, II or III evidence.

Table S3. Direction and Strength Ratings by Recommendations Across Clinical Practice Guidelines Included in Consistency Analysis.

Code	Recommendation Sources in Scope	Recommended			Not Recommended			Out of Scope
		Strong Strength	Weak Strength	Unspecified Strength	Weak Strength	Strong Strength	Unspecified Strength	
PIC-1	10	CMR (2021), EULAR (2020), SIR (2019), SFR (2019), CRA (2018), SIGN (2011), RACGP (2009)	APLAR (2019), HKSR (2019),	MoH Malaysia (2019), BSR (2012)				ACR (2021), GPCRCID (2020), NICE (2020)
PIC-2	2	SIR (2019), NICE (2020)						CMR (2021), GPCRCID (2020), ACR (2021), EULAR (2020), APLAR (2019), HKSR (2019), MoH Malaysia (2019), SFR (2019), CRA (2018), BSR (2012), SIGN (2011), RACGP (2009)
PIC-3	6	EULAR (2020), HKSR (2019), SIR (2019), SFR (2019), CMR (2021), CRA (2018)						ACR (2021), GPCRCID (2020), NICE (2020), APLAR (2019), MoH Malaysia (2019), BSR (2012), SIGN (2011), RACGP (2009)
PIC-4	5	EULAR (2020), HKSR (2019), SIR (2019), SFR (2019)	APLAR (2019)					ACR (2021), CMR (2021), GPCRCID (2020), NICE (2020), MoH Malaysia (2019), CRA (2018), BSR (2012), SIGN (2011), RACGP (2009)
PIC-5	5	EULAR (2020), HKSR (2019), SIR (2019), SFR (2019), CRA (2018)						ACR (2021), CMR (2021), GPCRCID (2020), NICE (2020), APLAR (2019), MoH Malaysia (2019), BSR (2012), SIGN (2011), RACGP (2009)

Code	Recommendation Sources in Scope	Recommended			Not Recommended			Out of Scope
		Strong Strength	Weak Strength	Unspecified Strength	Weak Strength	Strong Strength	Unspecified Strength	
PIC-6	5	EULAR (2020), HKSR (2019), SIR (2019), SFR (2019)	APLAR (2019)					ACR (2021), CMR (2021), GPCRCID (2020), NICE (2020), MoH Malaysia (2019), CRA (2018), BSR (2012), SIGN (2011), RACGP (2009)
PIC-7	2		NICE (2020)			HKSR (2019)		ACR (2021), CMR (2021), GPCRCID (2020), EULAR (2020), APLAR (2019), SIR (2019), MoH Malaysia (2019), SFR (2019), CRA (2018), BSR (2012), SIGN (2011), RACGP (2009)
PIC-8	2	EULAR (2020)	NICE (2020)					ACR (2021), CMR (2021), GPCRCID (2020), APLAR (2019), HKSR (2019), SIR (2019), MoH Malaysia (2019), SFR (2019), CRA (2018), BSR (2012), SIGN (2011), RACGP (2009)
PIC-9	2	HKSR (2019)	CRA (2018)					ACR (2021), CMR (2021), GPCRCID (2020), NICE (2020), EULAR (2020), APLAR (2019), SIR (2019), MoH Malaysia (2019), SFR (2019), BSR (2012), SIGN (2011), RACGP (2009)
PIC-10	3	EULAR (2020), HKSR (2019), SIR (2019)						ACR (2021), CMR (2021), GPCRCID (2020), NICE (2020), APLAR (2019), MoH Malaysia (2019), SFR (2019), CRA (2018), BSR (2012), SIGN (2011), RACGP (2009)
PIC-11	4	HKSR (2019), SIR (2019)	CRA (2018)					ACR (2021), CMR (2021), GPCRCID (2020), NICE (2020), EULAR (2020), APLAR (2019), MoH Malaysia (2019), SFR (2019), BSR (2012), SIGN (2011), RACGP (2009)
PIC-12	2	SIR (2019)	CRA (2018)					ACR (2021), CMR (2021), GPCRCID (2020), NICE (2020), EULAR (2020), APLAR (2019), HKSR (2019), MoH Malaysia (2019), SFR (2019), BSR (2012), SIGN (2011), RACGP (2009)

Code	Recommendation Sources in Scope	Recommended			Not Recommended			Out of Scope
		Strong Strength	Weak Strength	Unspecified Strength	Weak Strength	Strong Strength	Unspecified Strength	
PIC-13	2		EULAR (2020), SIR (2019)					ACR (2021), CMR (2021), GPCRCID (2020), NICE (2020), APLAR (2019), HKSR (2019), MoH Malaysia (2019), SFR (2019), CRA (2018), BSR (2012), SIGN (2011), RACGP (2009)
PIC-14	3	CMR (2021)	APLAR (2019)	MoH Malaysia (2019)				ACR (2021), GPCRCID (2020), NICE (2020), EULAR (2020), HKSR (2019), SIR (2019), SFR (2019), CRA (2018), BSR (2012), SIGN (2011), RACGP (2009)
PIC-15	4	EULAR (2020), SFR (2019), HKSR (2019)	ACR (2021)					CMR (2021), GPCRCID (2020), NICE (2020), APLAR (2019), SIR (2019), MoH Malaysia (2019), CRA (2018), BSR (2012), SIGN (2011), RACGP (2009)
PIC-16	3	EULAR (2020), SFR (2019)	ACR (2021)					CMR (2021), GPCRCID (2020), NICE (2020), APLAR (2019), HKSR (2019), SIR (2019), MoH Malaysia (2019), CRA (2018), BSR (2012), SIGN (2011), RACGP (2009)
PIC-17	2		EULAR (2020), SIR (2019)					ACR (2021), CMR (2021), GPCRCID (2020), NICE (2020), APLAR (2019), HKSR (2019), MoH Malaysia (2019), SFR (2019), CRA (2018), BSR (2012), SIGN (2011), RACGP (2009)
PIC-18	2		HKSR (2019), SIR (2019)					ACR (2021), CMR (2021), GPCRCID (2020), NICE (2020), EULAR (2020), APLAR (2019), MoH Malaysia (2019), SFR (2019), CRA (2018), BSR (2012), SIGN (2011), RACGP (2009)

Table S4. Consistency of Direction and Strength Ratings by Recommendation Across Clinical Practice Guidelines Excluding Unspecified Strength*.

**Code**	**Consistency of Direction**	**Consistency of Strength**	**Consistency of Direction (Excluding 'Unspecified Strength')**	**Consistency of Strength (Excluding 'Unspecified Strength')**	**Does excluding ' Unspecified Strength' make a difference in consistency of direction?**	**Does excluding ' Unspecified Strength' make a difference in consistency of strength?**
PIC-1	Yes	No	Yes	No	No	No
PIC-2	Yes	Yes	Yes	Yes	No	No
PIC-3	Yes	Yes	Yes	Yes	No	No
PIC-4	Yes	No	Yes	No	No	No
PIC-5	Yes	Yes	Yes	Yes	No	No
PIC-6	Yes	No	Yes	No	No	No
PIC-7	No	No	No	No	No	No
PIC-8	Yes	No	Yes	No	No	No
PIC-9	Yes	No	Yes	No	No	No
PIC-10	Yes	Yes	Yes	Yes	No	No
PIC-11	Yes	No	Yes	No	No	No
PIC-12	Yes	No	Yes	No	No	No
PIC-13	Yes	Yes	Yes	Yes	No	No
PIC-14	Yes	No	Yes	No	No	No
PIC-15	Yes	No	Yes	No	No	No
PIC-16	Yes	No	Yes	No	No	No
PIC-17	Yes	Yes	Yes	Yes	No	No
PIC-18	Yes	Yes	Yes	Yes	No	No

*Unspecified Strength: Recommended with unspecified strength of recommendation.

Table S5. Consistency of Direction and Strength Ratings by Recommendation Across Clinical Practice Guidelines Excluding ACR*.

**Code**	**Consistency of Direction**	**Consistency of Strength**	**Consistency of Direction (Excluding ' Unspecified Strength')**	**Consistency of Strength (Excluding ' Unspecified Strength')**	**Does excluding ' Unspecified Strength' make a difference in consistency of direction?**	**Does excluding ' Unspecified Strength' make a difference in consistency of strength?**
PIC-1	Yes	No	Yes	No	No	No
PIC-2	Yes	Yes	Yes	Yes	No	No
PIC-3	Yes	Yes	Yes	Yes	No	No
PIC-4	Yes	No	Yes	No	No	No
PIC-5	Yes	Yes	Yes	Yes	No	No
PIC-6	Yes	No	Yes	No	No	No
PIC-7	No	No	No	No	No	No
PIC-8	Yes	No	Yes	No	No	No
PIC-9	Yes	No	Yes	No	No	No
PIC-10	Yes	Yes	Yes	Yes	No	No
PIC-11	Yes	No	Yes	No	No	No
PIC-12	Yes	No	Yes	No	No	No
PIC-13	Yes	Yes	Yes	Yes	No	No
PIC-14	Yes	No	Yes	No	No	No
PIC-15	Yes	No	Yes	No	No	No
PIC-16	Yes	No	Yes	Yes	No	Yes
PIC-17	Yes	Yes	Yes	Yes	No	No
PIC-18	Yes	Yes	Yes	Yes	No	No

*ACR: American College of Rheumatology.

Table S6. Consistency of Direction and Strength Ratings by Recommendation Across Clinical Practice Guidelines Excluding CMR*.

Code	Consistency of Direction	Consistency of Strength	Consistency of Direction (Excluding ' Unspecified Strength')	Consistency of Strength (Excluding ' Unspecified Strength')	Does excluding 'Unspecified Strength' make a difference in consistency of direction?	Does excluding ' Unspecified Strength' make a difference in consistency of strength?
PIC-1	Yes	No	Yes	No	No	No
PIC-2	Yes	Yes	Yes	Yes	No	No
PIC-3	Yes	Yes	Yes	Yes	No	No
PIC-4	Yes	No	Yes	No	No	No
PIC-5	Yes	Yes	Yes	Yes	No	No
PIC-6	Yes	No	Yes	No	No	No
PIC-7	No	No	No	No	No	No
PIC-8	Yes	No	Yes	No	No	No
PIC-9	Yes	No	Yes	No	No	No
PIC-10	Yes	Yes	Yes	Yes	No	No
PIC-11	Yes	No	Yes	No	No	No
PIC-12	Yes	No	Yes	No	No	No
PIC-13	Yes	Yes	Yes	Yes	No	No
PIC-14	Yes	No	NA	NA	NA	NA
PIC-15	Yes	No	Yes	No	No	No
PIC-16	Yes	No	Yes	No	No	No
PIC-17	Yes	Yes	Yes	Yes	No	No
PIC-18	Yes	Yes	Yes	Yes	No	No

*CMR: Mexican College of Rheumatology.

Table S7. Consistency of Direction and Strength Ratings by Recommendation Across Clinical Practice Guidelines Excluding GPCRCID*.

Code	Consistency of Direction	Consistency of Strength	Consistency of Direction (Excluding ' Unspecified Strength')	Consistency of Strength (Excluding ' Unspecified Strength')	Does excluding ' Unspecified Strength' make a difference in consistency of direction?	Does excluding 'Unspecified Strength' make a difference in consistency of strength?
PIC-1	Yes	No	Yes	No	No	No
PIC-2	Yes	Yes	Yes	Yes	No	No
PIC-3	Yes	Yes	Yes	Yes	No	No
PIC-4	Yes	No	Yes	No	No	No
PIC-5	Yes	Yes	Yes	Yes	No	No
PIC-6	Yes	No	Yes	No	No	No
PIC-7	No	No	No	No	No	No
PIC-8	Yes	No	Yes	No	No	No
PIC-9	Yes	No	Yes	No	No	No
PIC-10	Yes	Yes	Yes	Yes	No	No
PIC-11	Yes	No	Yes	No	No	No
PIC-12	Yes	No	Yes	No	No	No
PIC-13	Yes	Yes	Yes	Yes	No	No
PIC-14	Yes	No	Yes	No	No	No
PIC-15	Yes	No	Yes	No	No	No
PIC-16	Yes	No	Yes	No	No	No
PIC-17	Yes	Yes	Yes	Yes	No	No
PIC-18	Yes	Yes	Yes	Yes	No	No

*GPCRCID: Guangdong Provincial Clinical Research Center for Immunological Diseases.

Table S8. Consistency of Direction and Strength Ratings by Recommendation Across Clinical Practice Guidelines Excluding NICE*.

**Code**	**Consistency of Direction**	**Consistency of Strength**	**Consistency of Direction (Excluding 'Unspecified Strength')**	**Consistency of Strength (Excluding 'Unspecified Strength')**	**Does excluding ' Unspecified Strength' make a difference in consistency of direction?**	**Does excluding 'Unspecified Strength' make a difference in consistency of strength?**
PIC-1	Yes	No	Yes	No	No	No
PIC-2	Yes	Yes	NA	NA	NA	NA
PIC-3	Yes	Yes	Yes	Yes	No	No
PIC-4	Yes	No	Yes	No	No	No
PIC-5	Yes	Yes	Yes	Yes	No	No
PIC-6	Yes	No	Yes	No	No	No
PIC-7	No	No	NA	NA	NA	NA
PIC-8	Yes	No	NA	NA	NA	NA
PIC-9	Yes	No	Yes	No	No	No
PIC-10	Yes	Yes	Yes	Yes	No	No
PIC-11	Yes	No	Yes	No	No	No
PIC-12	Yes	No	Yes	No	No	No
PIC-13	Yes	Yes	Yes	Yes	No	No
PIC-14	Yes	No	Yes	No	No	No
PIC-15	Yes	No	Yes	No	No	No
PIC-16	Yes	No	Yes	No	No	No
PIC-17	Yes	Yes	Yes	Yes	No	No
PIC-18	Yes	Yes	Yes	Yes	No	No

*NICE: National Institute of Health and Clinical Excellence.

Table S9. Consistency of Direction and Strength Ratings by Recommendation Across Clinical Practice Guidelines Excluding EULAR*.

**Code**	**Consistency of Direction**	**Consistency of Strength**	**Consistency of Direction (Excluding 'Unspecified Strength')**	**Consistency of Strength (Excluding 'Unspecified Strength')**	**Does excluding ' Unspecified Strength' make a difference in consistency of direction?**	**Does excluding ' Unspecified Strength' make a difference in consistency of strength?**
PIC-1	Yes	No	Yes	No	No	No
PIC-2	Yes	Yes	Yes	Yes	No	No
PIC-3	Yes	Yes	Yes	Yes	No	No
PIC-4	Yes	No	Yes	No	No	No
PIC-5	Yes	Yes	Yes	Yes	No	No
PIC-6	Yes	No	Yes	No	No	No
PIC-7	No	No	No	No	No	No
PIC-8	Yes	No	NA	NA	NA	NA
PIC-9	Yes	No	Yes	No	No	No
PIC-10	Yes	Yes	Yes	Yes	No	No
PIC-11	Yes	No	Yes	No	No	No
PIC-12	Yes	No	Yes	No	No	No
PIC-13	Yes	Yes	NA	NA	NA	NA
PIC-14	Yes	No	Yes	No	No	No
PIC-15	Yes	No	Yes	No	No	No
PIC-16	Yes	No	Yes	No	No	No
PIC-17	Yes	Yes	NA	NA	NA	NA
PIC-18	Yes	Yes	Yes	Yes	No	No

*EULAR: European League Against Rheumatism.

Table S10. Consistency of Direction and Strength Ratings by Recommendation Across Clinical Practice Guidelines Excluding APLAR*.

**Code**	**Consistency of Direction**	**Consistency of Strength**	**Consistency of Direction (Excluding ' Unspecified Strength')**	**Consistency of Strength (Excluding ' Unspecified Strength')**	**Does excluding 'Unspecified Strength' make a difference in consistency of direction?**	**Does excluding ' Unspecified Strength' make a difference in consistency of strength?**
PIC-1	Yes	No	Yes	No	No	No
PIC-2	Yes	Yes	Yes	Yes	No	No
PIC-3	Yes	Yes	Yes	Yes	No	No
PIC-4	Yes	No	Yes	Yes	No	Yes
PIC-5	Yes	Yes	Yes	Yes	No	No
PIC-6	Yes	No	Yes	Yes	No	Yes
PIC-7	No	NA	No	No	No	No
PIC-8	Yes	No	Yes	No	No	No
PIC-9	Yes	No	Yes	No	No	No
PIC-10	Yes	Yes	Yes	Yes	No	No
PIC-11	Yes	No	Yes	No	No	No
PIC-12	Yes	No	Yes	No	No	No
PIC-13	Yes	Yes	Yes	Yes	No	No
PIC-14	Yes	No	NA	NA	NA	NA
PIC-15	Yes	No	Yes	No	No	No
PIC-16	Yes	No	Yes	No	No	No
PIC-17	Yes	Yes	Yes	Yes	No	No
PIC-18	Yes	Yes	No	Yes	NA	NA

*APLAR: Asia-Pacific League of Associations for Rheumatology.

Table S11. Consistency of Direction and Strength Ratings by Recommendation Across Clinical Practice Guidelines Excluding HKSR*.

**Code**	**Consistency of Direction**	**Consistency of Strength**	**Consistency of Direction (Excluding ' Unspecified Strength')**	**Consistency of Strength (Excluding ' Unspecified Strength')**	**Does excluding 'Unspecified Strength' make a difference in consistency of direction?**	**Does excluding ' Unspecified Strength' make a difference in consistency of strength?**
PIC-1	Yes	No	Yes	No	No	No
PIC-2	Yes	Yes	Yes	Yes	No	No
PIC-3	Yes	Yes	Yes	Yes	No	No
PIC-4	Yes	No	Yes	Yes	No	Yes
PIC-5	Yes	Yes	Yes	Yes	No	No
PIC-6	Yes	No	Yes	Yes	No	Yes
PIC-7	No	NA	No	No	No	No
PIC-8	Yes	No	Yes	No	No	No
PIC-9	Yes	No	Yes	No	No	No
PIC-10	Yes	Yes	Yes	Yes	No	No
PIC-11	Yes	No	Yes	No	No	No
PIC-12	Yes	No	Yes	No	No	No
PIC-13	Yes	Yes	Yes	Yes	No	No
PIC-14	Yes	No	NA	NA	NA	NA
PIC-15	Yes	No	Yes	No	No	No
PIC-16	Yes	No	Yes	No	No	No
PIC-17	Yes	Yes	Yes	Yes	No	No
PIC-18	Yes	Yes	No	Yes	NA	NA

*HKSR: Hong Kong Society of Rheumatology.

Table S12. Consistency of Direction and Strength Ratings by Recommendation Across Clinical Practice Guidelines Excluding SIR*.

**Code**	**Consistency of Direction**	**Consistency of Strength**	**Consistency of Direction (Excluding 'Unspecified Strength')**	**Consistency of Strength (Excluding 'Unspecified Strength')**	**Does excluding ' Unspecified Strength' make a difference in consistency of direction?**	**Does excluding ' Unspecified Strength' make a difference in consistency of strength?**
PIC-1	Yes	No	Yes	No	No	No
PIC-2	Yes	Yes	NA	NA	NA	NA
PIC-3	Yes	Yes	Yes	Yes	No	No
PIC-4	Yes	No	Yes	No	No	No
PIC-5	Yes	Yes	Yes	Yes	No	No
PIC-6	Yes	No	Yes	No	No	No
PIC-7	No	No	No	No	No	No
PIC-8	Yes	No	Yes	No	No	No
PIC-9	Yes	No	Yes	No	No	No
PIC-10	Yes	Yes	Yes	Yes	No	No
PIC-11	Yes	No	Yes	No	No	No
PIC-12	Yes	No	NA	NA	NA	NA
PIC-13	Yes	Yes	NA	NA	NA	NA
PIC-14	Yes	No	Yes	No	No	No
PIC-15	Yes	No	Yes	No	No	No
PIC-16	Yes	No	Yes	No	No	No
PIC-17	Yes	Yes	NA	NA	NA	NA
PIC-18	Yes	Yes	NA	NA	NA	NA

*SIR: Italian Society for Rheumatology.

Table S13. Consistency of Direction and Strength Ratings by Recommendation Across Clinical Practice Guidelines Excluding MoH Malaysia*.

**Code**	**Consistency of Direction**	**Consistency of Strength**	**Consistency of Direction (Excluding 'Unspecified Strength')**	**Consistency of Strength (Excluding 'Unspecified Strength')**	**Does excluding 'Unspecified Strength' make a difference in consistency of direction?**	**Does excluding 'Unspecified Strength' make a difference in consistency of strength?**
PIC-1	Yes	No	Yes	No	No	No
PIC-2	Yes	Yes	Yes	Yes	No	No
PIC-3	Yes	Yes	Yes	Yes	No	No
PIC-4	Yes	No	Yes	No	No	No
PIC-5	Yes	Yes	Yes	Yes	No	No
PIC-6	Yes	No	Yes	No	No	No
PIC-7	No	No	No	No	No	No
PIC-8	Yes	No	Yes	No	No	No
PIC-9	Yes	No	Yes	No	No	No
PIC-10	Yes	Yes	Yes	Yes	No	No
PIC-11	Yes	No	Yes	No	No	No
PIC-12	Yes	No	Yes	No	No	No
PIC-13	Yes	Yes	Yes	Yes	No	No
PIC-14	Yes	No	Yes	No	No	No
PIC-15	Yes	No	Yes	No	No	No
PIC-16	Yes	No	Yes	No	No	No
PIC-17	Yes	Yes	Yes	Yes	No	No
PIC-18	Yes	Yes	Yes	Yes	No	No

*MoH Malaysia: Ministry of Health Malaysia.

Table S14. Consistency of Direction and Strength Ratings by Recommendation Across Clinical Practice Guidelines Excluding SFR*.

**Code**	**Consistency of Direction**	**Consistency of Strength**	**Consistency of Direction (Excluding 'Unspecified Strength')**	**Consistency of Strength (Excluding 'Unspecified Strength')**	**Does excluding ' Unspecified Strength' make a difference in consistency of direction?**	**Does excluding ' Unspecified Strength' make a difference in consistency of strength?**
PIC-1	Yes	No	Yes	No	No	No
PIC-2	Yes	Yes	Yes	Yes	No	No
PIC-3	Yes	Yes	Yes	Yes	No	No
PIC-4	Yes	No	Yes	No	No	No
PIC-5	Yes	Yes	Yes	Yes	No	No
PIC-6	Yes	No	Yes	No	No	No
PIC-7	No	No	No	No	No	No
PIC-8	Yes	No	Yes	No	No	No
PIC-9	Yes	No	Yes	No	No	No
PIC-10	Yes	Yes	Yes	Yes	No	No
PIC-11	Yes	No	Yes	No	No	No
PIC-12	Yes	No	Yes	No	No	No
PIC-13	Yes	Yes	Yes	Yes	No	No
PIC-14	Yes	No	Yes	No	No	No
PIC-15	Yes	No	Yes	No	No	No
PIC-16	Yes	No	Yes	No	No	No
PIC-17	Yes	Yes	Yes	Yes	No	No
PIC-18	Yes	Yes	Yes	Yes	No	No

*SFR: French Society for Rheumatology.

Table S15. Consistency of Direction and Strength Ratings by Recommendation Across Clinical Practice Guidelines Excluding CRA*.

**Code**	**Consistency of Direction**	**Consistency of Strength**	**Consistency of Direction (Excluding 'Unspecified Strength')**	**Consistency of Strength (Excluding 'Unspecified Strength')**	**Does excluding ' Unspecified Strength' make a difference in consistency of direction?**	**Does excluding ' Unspecified Strength' make a difference in consistency of strength?**
PIC-1	Yes	No	Yes	No	No	No
PIC-2	Yes	Yes	Yes	Yes	No	No
PIC-3	Yes	Yes	Yes	Yes	No	No
PIC-4	Yes	No	Yes	No	No	No
PIC-5	Yes	Yes	Yes	Yes	No	No
PIC-6	Yes	No	Yes	No	No	No
PIC-7	No	No	No	No	No	No
PIC-8	Yes	No	Yes	No	No	No
PIC-9	Yes	No	NA	NA	NA	NA
PIC-10	Yes	Yes	Yes	Yes	No	No
PIC-11	Yes	No	Yes	Yes	No	Yes
PIC-12	Yes	No	NA	NA	NA	NA
PIC-13	Yes	Yes	Yes	Yes	No	No
PIC-14	Yes	No	Yes	No	No	No
PIC-15	Yes	No	Yes	No	No	No
PIC-16	Yes	No	Yes	No	No	No
PIC-17	Yes	Yes	Yes	Yes	No	No
PIC-18	Yes	Yes	Yes	Yes	No	No

*CRA: Chinese Rheumatology Association.

Table S16. Consistency of Direction and Strength Ratings by Recommendation Across Clinical Practice Guidelines Excluding BSR*.

Code	Consistency of Direction	Consistency of Strength	Consistency of Direction (Excluding ' Unspecified Strength')	Consistency of Strength (Excluding ' Unspecified Strength')	Does excluding ' Unspecified Strength' make a difference in consistency of direction?	Does excluding ' Unspecified Strength' make a difference in consistency of strength?
PIC-1	Yes	No	Yes	No	No	No
PIC-2	Yes	Yes	Yes	Yes	No	No
PIC-3	Yes	Yes	Yes	Yes	No	No
PIC-4	Yes	No	Yes	No	No	No
PIC-5	Yes	Yes	Yes	Yes	No	No
PIC-6	Yes	No	Yes	No	No	No
PIC-7	No	No	No	No	No	No
PIC-8	Yes	No	Yes	No	No	No
PIC-9	Yes	No	Yes	No	No	No
PIC-10	Yes	Yes	Yes	Yes	No	No
PIC-11	Yes	No	Yes	No	No	No
PIC-12	Yes	No	Yes	No	No	No
PIC-13	Yes	Yes	Yes	Yes	No	No
PIC-14	Yes	No	Yes	No	No	No
PIC-15	Yes	No	Yes	No	No	No
PIC-16	Yes	No	Yes	No	No	No
PIC-17	Yes	Yes	Yes	Yes	No	No
PIC-18	Yes	Yes	Yes	Yes	No	No

*BSR: Brazilian Society of Rheumatology.

Table S17. Consistency of Direction and Strength Ratings by Recommendation Across Clinical Practice Guidelines Excluding SIGN*.

Code	Consistency of Direction	Consistency of Strength	Consistency of Direction (Excluding 'Unspecified Strength')	Consistency of Strength (Excluding 'Unspecified Strength')	Does excluding ' Unspecified Strength' make a difference in consistency of direction?	Does excluding ' Unspecified Strength' make a difference in consistency of strength?
PIC-1	Yes	No	Yes	No	No	No
PIC-2	Yes	Yes	Yes	Yes	No	No
PIC-3	Yes	Yes	Yes	Yes	No	No
PIC-4	Yes	No	Yes	No	No	No
PIC-5	Yes	Yes	Yes	Yes	No	No
PIC-6	Yes	No	Yes	No	No	No
PIC-7	No	No	No	No	No	No
PIC-8	Yes	No	Yes	No	No	No
PIC-9	Yes	No	Yes	No	No	No
PIC-10	Yes	Yes	Yes	Yes	No	No
PIC-11	Yes	No	Yes	No	No	No
PIC-12	Yes	No	Yes	No	No	No
PIC-13	Yes	Yes	Yes	Yes	No	No
PIC-14	Yes	No	Yes	No	No	No
PIC-15	Yes	No	Yes	No	No	No
PIC-16	Yes	No	Yes	No	No	No
PIC-17	Yes	Yes	Yes	Yes	No	No
PIC-18	Yes	Yes	Yes	Yes	No	No

*SIGN: Scottish Intercollegiate Guidelines Network.

Table S18. Consistency of Direction and Strength Ratings by Recommendation Across Clinical Practice Guidelines Excluding RACGP*.

**Code**	**Consistency of Direction**	**Consistency of Strength**	**Consistency of Direction (Excluding 'Unspecified Strength')**	**Consistency of Strength (Excluding 'Unspecified Strength')**	**Does excluding ' Unspecified Strength' make a difference in consistency of direction?**	**Does excluding 'Unspecified Strength' make a difference in consistency of strength?**
PIC-1	Yes	No	Yes	No	No	No
PIC-2	Yes	Yes	Yes	Yes	No	No
PIC-3	Yes	Yes	Yes	Yes	No	No
PIC-4	Yes	No	Yes	No	No	No
PIC-5	Yes	Yes	Yes	Yes	No	No
PIC-6	Yes	No	Yes	No	No	No
PIC-7	No	No	No	No	No	No
PIC-8	Yes	No	Yes	No	No	No
PIC-9	Yes	No	Yes	No	No	No
PIC-10	Yes	Yes	Yes	Yes	No	No
PIC-11	Yes	No	Yes	No	No	No
PIC-12	Yes	No	Yes	No	No	No
PIC-13	Yes	Yes	Yes	Yes	No	No
PIC-14	Yes	No	Yes	No	No	No
PIC-15	Yes	No	Yes	No	No	No
PIC-16	Yes	No	Yes	No	No	No
PIC-17	Yes	Yes	Yes	Yes	No	No
PIC-18	Yes	Yes	Yes	Yes	No	No

*RACGP: Royal Australian College of General Practitioners.
